# The Application of Artificial Neural Networks and Logistic Regression in the Evaluation of Risk for Dry Eye after Vitrectomy

**DOI:** 10.1155/2020/1024926

**Published:** 2020-04-21

**Authors:** Wan-Ju Yang, Li Wu, Zhong-Ming Mei, Yi Xiang

**Affiliations:** Department of Ophthalmology, The Central Hospital of Wuhan, Tongji Medical College of Huazhong University of Science and Technology, Wuhan 430014, Hubei Province, China

## Abstract

Supervised machine-learning (ML) models were employed to predict the occurrence of dry eye disease (DED) after vitrectomy in this study. The clinical data of 217 patients receiving vitrectomy from April 2017 to July 2018 were used as training dataset; the clinical data of 33 patients receiving vitrectomy from August 2018 to September 2018 were collected as validating dataset. The input features for ML training were selected based on the Delphi method and univariate logistic regression (LR). LR and artificial neural network (ANN) models were trained and subsequently used to predict the occurrence of DED in patients who underwent vitrectomy for the first time during the period. The area under the receiver operating characteristic curve (AUC-ROC) was used to evaluate the predictive accuracy of the ML models. The AUCs with use of the LR and ANN models were 0.741 and 0.786, respectively, suggesting satisfactory performance in predicting the occurrence of DED. When the two models were compared in terms of predictive power, the fitting effect of the ANN model was slightly superior to that of the LR model. In conclusion, both LR and ANN models may be used to accurately predict the occurrence of DED after vitrectomy.

## 1. Introduction

Vitrectomy, an ocular surgery performed to partially or completely remove the vitreous, is widely used to treat various ocular conditions, such as cloudy vitreous, vitreous haemorrhage, retinal detachment, and proliferative diabetic retinopathy [1–3]. Most vitrectomies are performed to facilitate surgery to address one of a variety of retinal conditions [3]. Some vitrectomies are conducted for diagnostic purposes. With advances in the instrumentation available, vitrectomy has become a well-established procedure. Serious vitrectomy-associated complications are very rare [1, 3]; however, like other types of ocular surgeries, vitrectomy may traumatize the conjunctival tissues, often resulting in the development of secondary dry eye disease (DED) [4–6]. Using the demographic and clinical features of patients to predict risk for vitrectomy-related DED will facilitate decision-making in the management of vitrectomy patients and improve the relationship between doctors and patients. To the best of our knowledge, no previous study has investigated the prediction of risk for secondary DED in patients scheduled to undergo vitrectomy.

In recent years, machine learning has been widely applied to solve real-life problems, including issues related to healthcare [7–9]. In supervised machine learning, the algorithm learns a target function from labelled training data. The outcome value of a case in unlabeled new data can be calculated based on the target function. The two learning tasks required for supervised learning are classification and regression. Although several techniques have been developed for supervised learning, those used most widely in healthcare and medicine are logistic regression (LR) and artificial neural networks (ANNs) [9, 10].

LR is a machine-learning technique borrowed from statistics. The logistic regression model, based on a logistic function, is used to express the relationship between multiple input features (independent variables) and a categorical dependent variable (outcome variable) and to predict the probability of a given outcome variable [8].

ANN is a nonlinear adaptive dynamic system that simulates biological nerve structure and consists of many processing units. It has become an important tool for predictive data applications [8]. In this study, we used logistic regression and an ANN to construct models for predicting the risk of secondary dry eye after vitrectomy. We evaluated the performance of these clinical prediction models in assessing the risk of secondary dry eye after vitrectomy in order to elucidate the mechanism of secondary dry eye after vitrectomy.

## 2. Materials and Methods

### 2.1. Patients

This study was approved by the Ethics Committee of Tongji Medical College of Huazhong University of Science and Technology. All procedures performed in studies involving human participants were in accordance with the ethical standards of the institutional and national research committee and with the 1964 Helsinki Declaration and its later amendments or comparable ethical standards. Written informed consent was obtained from all individual participants included in the study.

We retrospectively reviewed the data of patients who underwent vitrectomy in the Ophthalmology Department of our hospital during the period from January 1, 2014, to July 31, 2018; the data from these patients were used to train the supervised ML models. We also prospectively studied the patients who underwent vitrectomy during the period from January 1, 2018, to September 1, 2018. The datasets from these patients were used to validate the ML models.

The inclusion criteria for enrolment for training and validation of the ML models were as follows: (1) complete clinical data and clear outcomes; (2) age ≥18 years and ability to articulate one's feelings; (3) initial presentation at our institution; (4) history of complete vitrectomy; and (5) targeted diagnosis and treatment for the initial medical concern.

The exclusion criteria included the following: (1) a voluntary request from the patient to terminate treatment during the perioperative period, followed by early discharge; (2) previous diagnosis with xerophthalmia (with or without treatment); (3) previous history of complications such as acute conjunctivitis, glaucoma, keratitis, ocular trauma, dacryocystitis, and systemic lupus erythematosus; (4) history of contact lens wear; (5) history of laser or other eye operations; (6) history of disease requiring long-term use of atropine, neostigmine, artificial tears, or other drugs that affect tear film stability; and (7) refusal to cooperate with the necessary examinations.

### 2.2. Diagnosis of Dry Eye Disease

DED was diagnosed according to the guidelines provided in the TFOS DEWS II diagnostic methodology report [11]. These were as follows: (1) screening questionnaire scored >5 or OSDI >13, accompanied by noninvasive break-up time (BUT) ≤10; (2) osmolarity >308 mOsm/L or interocular difference >8 mOsm/L; and (3) ocular surface staining >5 corneal spots or >9 conjunctival spots, or lid margin ≥2 mm in length and ≥25 mm in width.

### 2.3. Feature Selection for ML

Four representative ophthalmologists screened randomized patients with the Delphi method. Each patient was screened twice. The potentially relevant factors that were treated as categorical variables were gender, age, history of hypertension, history of diabetes mellitus, history of smoking, indoor work, occupation, daily exposure to computer or mobile phone screens, and driving conditions (driving time per day).

For the preoperative Schirmer I test (SIT), 5 mm filter paper was placed at a point 1/3 of the length of the lower conjunctival sac with respect to the medial canthus in the absence of ocular surface anesthesia. After the patient had gently closed his/her eyes for 5 minutes, the filter paper was taken out and the length of the wet filter paper was measured from the fold. To quantify preoperative BUT, sodium fluorescein solution was dripped into the conjunctival sac. After the patient had blinked several times, he/she was asked to look straight ahead. The patient was evaluated under wide-angle cobalt blue light from the slit lamp. The time from the last blink to appearance of the first black spot on the cornea was considered as tear film rupture time. After repeated measurements, the average value was obtained. For preoperative corneal fluorescein staining, fluorescein solution was dripped into the conjunctival sac of the eye that had been operated upon, which was then observed under the cobalt blue light from the slit lamp. The presence of any corneal epithelial defect was recorded. The range of corneal staining was scored as follows: corneal epithelial nonstaining, 0; dispersed fluorescence throughout the cornea, 1; slightly dense corneal staining, 2; and dense or flaky corneal staining, 2. Intraocular pressure (IP) was measured with a noncontact tonometer, with the range of normal values considered to be 10–21 mmHg. Corneal central thickness (CCT) of the operated eye was determined with an Orbscan II anterior segment analyzer.

Use of the Delphi method for analysis dictated exclusion of the following factors: history of hyperlipidemia, drinking history, educational background, correct reading posture, body mass index (BMI), duration of disease, proximity of contaminated buildings, long-term exposure to air-conditioning, and daily use of a mobile phone postoperatively.

### 2.4. Machine-Learning Construction and Testing

#### 2.4.1. LR Model

Univariate analysis was performed to determine the regression coefficient for each potential influencing factor. The variables revealed to have statistical significance after univariate analysis were input to train the multivariate logistic regression model to establish the prediction equation. Stepwise regression analysis was used to eliminate variables for modeling and to observe whether there were statistical differences between variables in the goodness of fit. The Wald-2 test was performed to estimate the logistic regression equation and regression coefficient. The partial regression coefficient (B), standard error (S.E.), Wald statistics, and *p* value were obtained for the corresponding variables, and the multivariate logistic regression equation was constructed.

#### 2.4.2. ANN Model

The ANN model was used to analyze the relationship between secondary DED, diagnosed 3 months after surgery, and to identify potential risk factors for vitrectomy-associated DED. The neural network model was set using a multilayer perceptron neural network. The numbers of hidden layers and network neurons were automatically determined by network optimization. First, factors thought to increase risk for dry eye secondary to vitrectomy were extracted as input layer vectors. Second, we established a neural network model, which consisted of three layers: input and output layers on both sides and hidden layers in the middle. Each hidden layer comprised multiple layers. Finally, forward and backward propagation networks were trained. For forward propagation, independent variables were input into the neural network from the input layer and then passed through several hidden layers. Finally, the prediction results were output to the output layer. For back propagation, an error backpropagation algorithm and the gradient descent optimization method were used to adjust the weights of each network layer. Error information was obtained by comparing the output information and expected information. The chain derivation method was employed to obtain error information for each step. Each layer was propagated forward to obtain the corresponding error information, and the weight and bias of each layer were adjusted accordingly.

ANN training and validation were carried out using MATLAB 2012 software. The network type was feedforward backpropagation; the training function was trainlm; the learning function was learngdm; and the error performance function was mse. The tansig function was used to complete the transfer of each layer, and the training times were set at 1000.

Finally, the predictions obtained with the methods described above were used as test variables. Actual prognosis outcomes were used as state variables; 1-specificity was used as the abscissa; and sensitivity was used as the ordinate. Then, the receiver operating characteristic curve (ROC curve) was drawn, and the area under the curve (AUC) and 95% CI were calculated. The binormal model was fitted according to the data results; the corresponding parameters were estimated with the maximum likelihood method; and the smooth ROC curve was obtained. The ROC curve was used to test the ML models in order to further clarify the impact value of each factor on the outcome.

### 2.5. Statistical Analysis

Data analyses were conducted using the SPSS 21.0 software package, and differences were considered statistically significant when *p* < 0.05. The Kolmogorov–Smirnov test was used for the measurement data, and measurement data conforming to the normal distribution were expressed as mean (+SD/SEM). The independent sample *t*-test was used for between-group comparisons. Single-factor analysis of variance was used for multiple-group comparisons. Medians (*M*) and quartiles (Q25 and Q75) were used for measurement data that did not conform to the normal distribution.

## 3. Results

### 3.1. Patients and Datasets for Training and Validation

After screening, 217 cases of vitrectomy that satisfied the study's inclusion and exclusion criteria were ultimately obtained, including 57 cases of giant retinal detachment, 42 cases of cataract with retinal detachment, 38 cases of proliferative diabetic retinopathy, 33 cases of idiopathic macular hole, 30 cases of exudative retinal detachment, and 17 cases of acute retinal necrosis syndrome. The average hospitalization time was 7.26 days. On the 10th day, 36 patients were diagnosed with secondary DED.

The validation dataset included clinical data for 33 cases of vitrectomy: 21 cases of giant retinal detachment, 9 cases of cataract complicated with retinal detachment, and 3 cases of endophthalmitis. The average hospitalization time was 8.76 (±1.28 days). Five cases had vitrectomy-associated DED.

### 3.2. LR Analysis for Predicting Risk for Vitrectomy-Associated DED

Univariate analysis was performed with the chi-square test and the independent sample *t*-test. The factors correlated with secondary dry eye after surgery were gender (male), age, history of diabetes mellitus, history free of smoking, smoking more than 10 cigarettes per day, indoor work, daily exposure to computer and mobile phone preoperatively, preoperative BUT, and preoperative CCT (*P* < 0.05; Table 1).

The dependent outcome variable, presence or absence of vitrectomy-associated DED, was binary. Variables associated with significant differences in univariate analysis were used as input features to train the logistic regression model by stepwise regression analysis. Significant differences between variables in goodness of fit were observed. The Wald-2 test was performed to estimate the logistic regression equation and the regression coefficient. The final independent influencing factors (all were risk factors), in order from most to least important, were age, history of diabetes mellitus, smoking more than 10 cigarettes per day, daily exposure to electronic screens preoperatively, preoperative BUT, and duration of surgery (*P* < 0.05; Table 2). The goodness-of-fit test of the multivariate logistic regression equation showed that *χ*2 = 8.083, DF = 7, and *P*=0.374, suggesting satisfactory goodness-of-fit. The equation was as follows:

(1) $$\begin{array}{c} P=\frac{1}{1+0.240\left[-\left(1.612+0.753X_{2}+0.623X_{3}+1.130X_{4}+1.112X_{6}+0.286X_{7}+0.889X_{9}\right)\right]}, \end{array}$$

where X_2_ is age; X_3_ is history of diabetes mellitus; X_4_ is smoking more than 10 cigarettes per day; X_6_ is daily exposure to computer or mobile phone screen; X_7_ is preoperative BUT; and X_9_ is duration of surgery.

### 3.3. Predictive Accuracy of the LR Model

**Figure 1:**
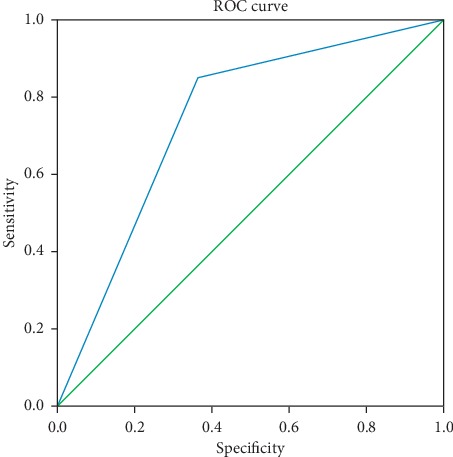
ROC curve for evaluating the performance of the logistic regression model in predicting the occurrence of dry eye disease.

We substituted specific values for the independent factors from the validation dataset into the formulas presented above. We compared the outcomes predicted by these formulas with the actual outcomes and then evaluated the predictive accuracy of the LR model. The performance of the LR model was tested by the ROC curve and showed AUC = 0.741, 95% CI = 0.611–0.870, and *P* < 0.05. These findings suggest that the prediction model was effective in predicting the occurrence of postoperative DED secondary to vitrectomy (Figure 1).

### 3.4. ANN Model and Its Performance in Predicting Vitrectomy-Associated DED

The neural network of multilayer perceptron was enhanced. The numbers of layers and neurons in hidden layers were determined automatically by network optimization. Potential influencing factors related to vitrectomy were used as input variables for the network model, with occurrence of vitrectomy-associated DED as output variable. As shown above, 217 subjects and 33 subjects were included in the training and test datasets, respectively. Analysis of the artificial neural network identified the following as influencing factors (independent variables) correlated with DED secondary to vitrectomy (in order from most to least important): age (100%), daily exposure to computer or mobile phone screen preoperatively (76.93%), preoperative BUT (69.18%), preoperative CCT (65.24%), and daily smoking (>10) (62.69%). The ANN model is summarized in Table 3.

**Figure 2:**
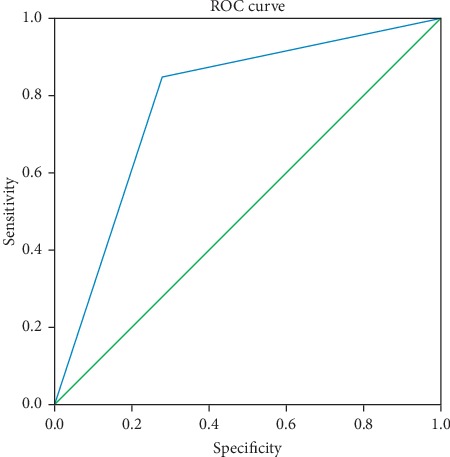
ROC curve for evaluating the performance of the ANN model in predicting the occurrence of dry eye disease.

The trained ANN model was used to test the validating dataset. The classification of the ANN model is shown in Table 4 via comparison of predicted vs. actual outcomes. As shown in Figure 2, the performance of the ANN model in predicting the occurrence of DED was tested by the ROC curve, with the following parameters: AUC = 0.786, 95% CI = 0.667–0.906, and *P* < 0.05. The ROC curve showed that the prediction model was effective for the prediction of secondary DED after vitrectomy.

### 3.5. Comparison of the LR and ANN Models in terms of Predictive Accuracy

The ROC curves for the two models tested showed that the predictive accuracy of the ANN model (AUC = 0.786) was slightly better than that of the LR model (AUC = 0.741).  Table 5 shows the detailed parameters of the ROC curves for both ML models.

## 4. Discussion

This is the first study to use supervised ML models to predict the risk of DED after vitrectomy. Both LR and ANN models were trained with the labelled data retrieved from previous cases of vitrectomy. Both models performed similarly in predicting the occurrence of DED, but the ANN model performed slightly better than the LR model.

The results of this study demonstrate that the LR model and ANN model had similar predictive accuracy. The AUC of the ROC curve were 0.741 and 0.786, respectively, suggesting that the performance of the ANN model is slightly better than that of the LR model. Notably, both models identified age as the top risk factor for vitrectomy-associated DED. In addition, both LR and ANN models identified four common independent risk factors for DED after vitrectomy: age, smoking more than 10 cigarettes per day, daily exposure to computer or mobile phone screens preoperatively, and preoperative BUT. History of diabetes mellitus and surgical duration were identified as risk factors only by the LR model, while preoperative CCT was only identified as a risk factor for vitrectomy-associated DED by the ANN model. LR performs better for qualitative and semiquantitative (multiclassification) independent variables, while ANNs use either categorical or continuous variables as input. These facts may explain the differences between the results provided by these two models. This indicates that the ANN model has superior predictive adaptability for use in clinical research.

Few studies have investigated the influencing factors that affect the occurrence of DED after vitrectomy. Our results are consistent with previous reports that age and surgical duration are risk factors for vitrectomy-related DED [12]. In addition, Banaee et al. (2008) reported that scleral depression significantly increased risk for DED after vitrectomy [5].

The tear film, which comprises lipid, tear, and mucin layers, nourishes the conjunctival epithelium and cornea, supplies lubrication to facilitate opening and closing of the eyelids, and provides a high-quality optical surface for the cornea [13, 14]. The pathogenesis of DED includes inflammation, apoptosis of the lacrimal gland cells and conjunctival epithelial cells, and androgen imbalance [13, 14]. The results of this study helped us to identify the mechanisms underlying DED after vitrectomy. First, the corneal epithelium and conjunctiva may be damaged by vitrectomy. After the operation, numerous factors may disturb the ocular surface, including scleral sutures, conjunctival sutures, incisions, and conjunctival edema. Corneal curvature may be affected, resulting in a decrease in tear film stability [15]. Importantly, it has been reported that basic fibroblast growth factor (alone or in combination with cytochrome c peroxidase) accelerates the healing of surgically damaged corneal epithelium [16, 17]. We therefore sought to investigate the benefit of reducing the occurrence of DED in patients who had undergone vitrectomy. Second, vitrectomy-associated congestion and edema of the corneal tissue may affect the adhesion of mucin, allowing for the infiltration of inflammatory factors. This process can cause lacrimal gland damage, which exacerbates any corneal damage [18]. Prolonged surgical time can thus destroy the stability of the tear film and lead to secondary dry eye after vitrectomy. Third, corneal goblet cells are more sensitive and vulnerable to external environmental factors, such as hyperglycemia (in diabetic patients). Metabolic disorders and nutritional disorders shorten the tear film rupture time and destroy the normal corneal morphology, thereby reducing the secretion of mucin. Finally, the eye drops often prescribed for patients after vitrectomy contain preservatives, which can affect corneal epithelial integrity, damage repair functions, and reduce the regularity of the corneal surface. All of these factors decrease tear film stability [19].

The analysis of clinical research data is challenging due to the complexity involved: on one hand, data need to meet the constraints of analytical models; on the other hand, the data characteristics need to be retained as far as possible in order to simulate the clinical situation [20]. It is therefore of great clinical significance to use the limited clinical data available for patients who have undergone vitrectomy for data analysis. Such a data-based approach will improve the data model for predicting secondary DED after vitrectomy and help physicians to identify risks early enough to communicate effectively with patients and to provide pertinent clinical interventions.

Logistic/Cox regression analysis is suitable for discriminating two or more classified variables, obtaining approximate estimates of relative risk and calculating their respective probabilities. Logistic/Cox regression analysis can be used to analyze most clinical data, but the flexibility and ease of use are ineffective for processing multiclass clinical data. With the increasingly close integration of computer science and applied mathematics with clinical medicine, more and more analytical and computational tools have been applied to clinical research. Various problems encountered by those performing clinical data analysis have been solved.

As a digital model which imitates the functional structure of a biological neural network, the ANN model utilizes large-scale nonlinear parallel processing and strong adaptability. The ANN model does not restrict the distribution of data, allowing researchers to make full use of data information. The ANN model has strong fault tolerance, so it can be widely used in the fields of prediction and analysis [21]. The ANN model also has better fit than the LR model. The identification of risk factors for vitrectomy-associated DED and the accurate prediction of secondary DED after vitrectomy by ML models will be helpful for clinical decision-making, as well as the management of patients who have undergone vitrectomy.

## 5. Conclusions

In conclusion, our study has shown that the LR and ANN models are similarly effective in predicting the occurrence of DED after vitrectomy. However, the ANN model better reflects the true relationship between input variables and the outcome variable.

## Figures and Tables

**Table 1:** Univariate LR analysis of dry eye disease after vitrectomy.

Groups	Without dry eye (*n* = 181)	With dry eye (*n* = 36)	*p* value
Male, *n* (%)	119 (65.75)	16 (44.44)	0.020^*∗*^
Age (years)	46 (29, 70)	54 (38, 72)	0.018^*∗*^
Hypertension, *n* (%)	47 (25.97)	9 (25.00)	0.417
Diabetes, *n* (%)	10 (5.52)	15 (30.56)	0.009^*∗*^
Smoking			
none, *n* (%)	122 (67.40)	19 (52.78)	0.040^*∗*^
＜10, *n* (%)	32 (17.68)	7 (19.44)	0.203
≥10, *n* (%)	27 (14.92)	10 (27.78)	0.042^*∗*^
Indoor work, *n* (%)	113 (62.43)	30 (83.33)	0.018^*∗*^
Occupations			
Farmer, *n* (%)	9 (4.97)	2 (5.56)	0.415
Worker, *n* (%)	23 (12.71)	4 (11.11)	0.338
White collar, *n* (%)	58 (32.04)	11 (30.56)	0.316
Student, *n* (%)	20 (11.05%)	4 (11.11)	0.556
Others, *n* (%)	40 (22.10)	9 (25.00)	0.289
Unemployed, *n* (%)	31 (17.13)	6 (16.67)	0.334
Preoperative daily use of computer and smart phone (*h*)	6 (2, 11)	10 (5, 14)	0.018^*∗*^
Preoperative daily driving time			
None, *n* (%)	79 (43.65)	15 (41.67)	0.258
＜1 h, *n* (%)	41 (22.65)	9 (25.00)	0.189
≥1 h, *n* (%)	61 (33.70)	12 (33.33)	0.486
Preoperative Sit (mm)	13.03 ± 4.70	12.85 ± 3.28	0.181
Preoperative BUT (s)	11.25 ± 2.24	8.72 ± 1.29	0.039^*∗*^
Preoperative CFS (min)	2.85 ± 0.77	2.48 ± 0.65	0.248
Preoperative IP (mmHg)	9.85 ± 1.74	11.05 ± 2.12	0.088
Preoperative CCT (mm)	0.525 ± 0.021	0.637 ± 0.025	0.017^*∗*^
Light intensity during the operation			
Low, *n* (%)	43 (23.76)	10 (27.78)	0.206
Moderate, *n* (%)	111 (61.33)	21 (58.33)	0.184
High, *n* (%)	27 (14.92)	5 (13.89)	0.320
Surgical duration (min)	56.35 ± 12.26	77.89 ± 10.78	0.021^*∗*^
Corneal protection during operation, *n* (%)	164 (90.60)	22 (70.98)	0.026^*∗*^

^*∗*^ *P* < 0.05; Sit, Schirmer I test; BUT, break-up time; CFS, corneal fluorescein staining; IP, intraocular pressure; CCT, corneal central thickness.

**Table 2:** Multivariate logistic regression analysis of dry eye disease after vitrectomy.

Groups	B	S.E.	Wald χ^2^	OR	95% CI	*p* value
Male	−0.202	0.009	1.871	0.595	0.289–1.193	0.146
Age	0.753	0.081	3.659	1.254	1.081–1.708	0.039^*∗*^
Diabetes	0.623	0.122	4.481	2.028	1.481–3.289	0.018^*∗*^
Smoking						
No	—	—	1.157	—	—	0.233
<10	−0.890	0.198	1.002	0.894	0.449–1.215	0.175
≥10	1.130	0.074	3.589	1.436	1.084–2.158	0.041^*∗*^
Indoor work	0.440	0.047	1.226	1.450	0.801–2.130	0.137
Preoperative daily use of computer and smartphone	1.122	0.320	5.701	2.156	1.707–3.008	0.019^*∗*^
Preoperative BUT	0.286	0.049	3.791	1.430	1.119–1.951	0.036^*∗*^
Preoperative CCT	0.225	0.065	1.844	1.289	0.887–1.578	0.202
Surgical duration	0.889	0.120	4.250	1.980	1.336–3.265	0.012^*∗*^

Constant	0.805	0.104	5.884	1.612	—	0.020^*∗*^

^*∗*^ *P* < 0.05.

**Table 3:** Summary of the artificial neural network analysis.

Datasets	Summary	
Training	SSE	51.849
	Percent error prediction	15.62
	Stopping rule	Error calculation based on test sample
Training time (s)		78
Validating	SSE	35.817
	Percent error prediction	13.36

**Table 4:** Classification by the artificial neural network model.

Datasets	Observed	Predicted	
		Without dry eye	With dry eye
Training	Without dry eye	161	20
	With dry eye	20	16
Validation	Without dry eye	26	2
	With dry eye	2	3

**Table 5:** Comparison of predictive accuracy between the logistic regression model and the artificial neural network model.

Groups	AUC	95% CI		*P* value	Critical points	
		Minimum	Maximum		Sensitivity (%)	Specificity (%)
Logistic regression	0.741	0.611	0.870	0.006^*∗*^	81.9	62.0
ANN	0.786	0.667	0.906	0.001^*∗*^	83.1	72.4

^*∗*^ *P* < 0.05.

## Data Availability

The datasets generated and analyzed during the present study are available from the corresponding author upon reasonable request.
